# Comparison of the classifiers based on mRNA, microRNA and lncRNA expression and DNA methylation profiles for the tumor origin detection

**DOI:** 10.3389/fgene.2024.1383852

**Published:** 2024-06-12

**Authors:** Yun Feng, Yilin Wang

**Affiliations:** Department of Hepatic Surgery, Fudan University Shanghai Cancer Center, Shanghai Medical College, Fudan University, Shanghai, China

**Keywords:** gene expression profile, DNA methylation profile, tumor tissue origin detection, machine learning, cancer of unknown primary

## Abstract

**Background:**

Tumor tissue origin detection is of great importance in determining the appropriate course of treatment for cancer patients. Classifiers based on gene expression and DNA methylation profiles have been confirmed to be feasible and reliable to predict the tumor primary. However, few works have been performed to compare the performance of these classifiers based on different profiles.

**Methods:**

Using gene expression and DNA methylation profiles from The Cancer Genome Atlas (TCGA) project, eight machine learning methods were employed for the tumor tissue origin detection. We then evaluated the predictive performance using DNA methylation, mRNA, microRNA (miRNA) and long non-coding RNA (lncRNA) expression profiles in a comparative manner. A statistical method was introduced to select the most informative CpG sites.

**Results:**

We found that LASSO is the most predictive models based on various profiles. Further analyses indicated that the results derived from DNA methylation (overall accuracy: 97.77%) are better than those derived from mRNA expression (overall accuracy: 88.01%), microRNA expression (overall accuracy: 91.03%) and lncRNA expression (overall accuracy: 95.7%). It has been suggested that we can achieve an overall accuracy >90% using only 1,000 methylated CpG sites for prediction.

**Conclusion:**

In this work, we comprehensively evaluated the performance of classifiers based on different profiles for the tumor origin detection. Our findings demonstrated the effectiveness of DNA methylation as biomarker for tracing tumor tissue origin using LASSO and neural network.

## Introduction

Metastatic cancer of unknown primary (CUP) origin accounts for about 3%–5% of all cancer diagnoses (Pimiento et al., 2007). Patients with CUP origin are always associated with poor prognosis because of late diagnosis, and even worse, some patients may be misclassified for tumor tissue origin. Despite the development of diagnostic workups, they show relatively little benefit (Hainsworth and Greco, 1993; Oien, 2009). In this regard, it is necessary to find new strategies to improve diagnostic certainty, and the ability to identify tumor tissue origin holds great promise for improving prognosis and treatment selection.

Molecular characterization is increasingly used for cancer therapy and offers great potential for tumor diagnosis (Tothill et al., 2005; Wang et al., 2015). Cancer classification based on expression profiles was introduced and has been generally proposed as a clinical application for tumor tissue origin detection (Ramaswamy et al., 2001; Bloom et al., 2004; Staub et al., 2010). The rationale using ‘-omics’ data to define the origin site of CUP is that tumors from different sites of origin have specific expression profile (Pimiento et al., 2007; Sotiriou and Piccart, 2007; Xu et al., 2016; Zheng et al., 2018). More importantly, gene expression profiling enables the measurement of expression levels of thousands of genes in a single experiment. For example, mRNA-based classifier was used to determine the CUP origin and the classifier achieved an accuracy of 89% (Tothill et al., 2005). A 92-gene qRT-PCR assay has been developed to detect the site of origin of metastatic tumors (Ma et al., 2006). MicroRNA can regulate gene expression and showed marked tissue specificity (Lagos-Quintana et al., 2002; Babak et al., 2004; Lu et al., 2005; Yang et al., 2017). The expression profiles of microRNAs have been determined in paraffin-embedded samples, and machine learning based classifiers achieved competitive performance (Rosenfeld et al., 2008; Varadhachary et al., 2011). DNA methylation is an epigenetic mechanism used by cells to control gene expression, which can fix genes in the “off” position (Ehrlich, 2002; Paz et al., 2003; Schubeler, 2015). Extensive DNA methylation perturbation have been widely explored in human cancer researches (Moran et al., 2016; Hao et al., 2017; Kang et al., 2017; Shen et al., 2017; Stieglitz et al., 2017). These works suggested that DNA methylation might be an additional way to help tumor tissue origin detection.

To comprehensively evaluate the potential and limitation of utilizing different profiles, we performed tumor tissue origin detection using eight different classification machine learning models (random forest, support vector machine, K-nearest neighbor, decision tree, linear discriminant analysis, LASSO, artificial neural network, naïve Bayesian classifier) and evaluated the predictive performance of these models in a comparative manner. These works reinforced the potential of DNA methylation as biomarkers for tumor tissue origin detection.

## Materials and methods

### Data collection

Cancer gene expression (mRNA, miRNA and lncRNA) and DNA methylation profiles generated by the Cancer Genome Atlas (TCGA) project were downloaded via the cBioPortal for Cancer Genomics (Cerami et al., 2012). For TCGA gene expression and DNA methylation data, only level one data was employed in our analysis. RNA-SeqV2 was used, which takes transcript length into account and is suggested to provide more accurate results. This work only contains the data of solid tumors, and a data quality control were conducted. For each cancer type, the dataset should have a sufficient number of samples (>100). The DNA methylation profiles were measured by the Infinium HumanMethylation450 platform, and we removed those CpG sites with more than 30% missing sample values. The remaining missing values were calculated using the K-nearest neighbor method. In this work, we adopted the same cohort for each dataset, and a total of 6,738 tumor samples for mRNA, miRNA, lncRNA and DNA methylation-based profile were collected spanning 20 cancer types.

### Classifiers construction

In this work, we employed eight machine learning classifiers for tumor tissue origin detection (Rosenfeld et al., 2008; Moran et al., 2016; Hao et al., 2017; Soh et al., 2017; Tang et al., 2017). These methods differ in their underlying methodology, and detailed descriptions of these models appear below. All these models were implemented in Python packages (v3.9).

*Random forest* (RF) is an ensemble learning algorithm for classification that works based on a multitude of decision trees. Each tree in the forest is built from a sample set drawn from the training set with replacement. Each feature used to split an internal node in the decision trees are picked from a random subset of the entire feature set. We used the soft voting strategy, i.e., the probabilities assigned to each class are calculated by averaging the output of each decision tree. The number of trees are set to 200.

*Support vector machine* (SVM) classifier is used to find a hyper-plane to separate two classes through maximizing the distance between the hyper-plane and the support vectors, which are defined as the samples closest to the hyper-plane. For those which are not separable, SVM is able to classify them through mapping the points into a higher dimensional space. In this work, the linear kernel was used. For multi-class classification, we implemented the ‘One-vs-Rest’ (OvR) approach.

*K-nearest neighbor* (KNN) is a non-metric method for classification. KNN simply saves all the samples in training set. Each time a test sample is given, KNN calculates the distances between the sample and all the data points in the training set. The test sample is classified as the class most common among its k nearest neighbors. Here, we used Euclidean distance, and set K to 5.

*Decision trees* (DT) are tree-like models used for classification. Each internal node within a decision tree represents a classification rule and each leaf node represents a class label. We built Classification and Regression Trees (CART), which choose features through minimizing the Gini index at each node.

*Least Absolute Shrinkage and Selector Operator* (LASSO) is a linear classification model that uses L1-regularization strategy in parameter estimation. The probabilities of each class are calculated via logistic function. To avoid over fitting, the one norm of the coefficient was added in the loss function, and coefficients were calculated through minimizing the loss function.

*Neural network* (NN) is based on a collection of connected nodes called neurons. Each neuron receives the input signals of other neurons through weighted connection, and produces output through activation function. If the weighted sum of input signals exceeds a cutoff, the neuron will be activated and outputs a non-zero value. Here we use the rectified linear unit function (ReLU) as the activation function. We used multi-layer feed-forward neural network, where all the neurons are connected with the next layer, and neurons within a layer are not connected with each other. The network was trained using error BackPropagation (BP) algorithm.

*Naïve Bayesian classifier* (NBC) is a classifier based on Bayes’ theorem and the assumption of independence among all features. Assuming that all feature is independent, the joint distribution equals the multiplication of marginal distribution. The probabilities assigned to each class is calculated through Bayes’ theorem, and the predicted class is the class with the largest probability value.

*Linear discriminant analysis* (LDA) is a linear model used in classification. Given a dataset with two classes, LDA projects all the sample points to a line, trying to maximize the distance of the centers of two classes and minimize the dispersion of points within the same class. The covariance matrix is used to measure the dispersion within a class. Here, we took ‘One-vs-Rest’ (OvR) strategy to construct multi-classifier.

### Performance evaluation

We compared the predictive performance of these models by tracing their overall accuracy. Overall accuracy measures how often a machine learning model correctly predicts the outcome. We calculated the overall accuracy by dividing the number of correct predictions by the total number of predictions. To further evaluate our models, 5-fold cross-validation was performed. Briefly, we randomly divided the data into five sets with approximately equal size, and used four of the five sets as the training set and the remaining set as the testing set to identify the positives and negatives. We considered precision and recall for specific cancer type *i*:

$${\text{Precision}}_{i}=\frac{Number\;of\;samples\;correctly\;classified\;as\;cancer\;type\;i}{Number\;of\;samples\;classified\;as\;cancer\;type\;i}$$

$${\text{Recall}}_{i}=\frac{Number\;of\;samples\;correctly\;classified\;as\;cancer\;type\;i}{Number\;of\;samples\;of\;cancer\;type\;i}$$

### Dimensionality reduction

Due to the high dimensionality of DNA methylation profiles, the dimensionality reduction step is necessary before the classifier construction. Principle component analysis (PCA) is a statistical procedure to reduce the dimensionality of a dataset with a large number of interrelated variables by creating a new set of variables called principal components. The greatest variance by some projection of the data comes to lie on the first coordinate (*w*1), the second greatest variance on the second coordinate (*w*2), and so on. The principal components were selected based on cumulative percentage of total variations. We selected the number of principle of components taken together explaining more than 95% of the variance.

### Feature selection using DNA methylation

At first, we identified tissue-specific DNA methylated sites to reduce the considerable redundancy of the original data. We calculated differential methylation values (*β* value) of CpGs for the corresponding cancer type compared with other cancers using Student’s t-test with a threshold of *F.D.R.* < 0.01. Next, a recent proposed feature selection method was employed, named Maximum-F-statistic-Maximum-Distance (MFMD), to further detect the tissue-specific CpG sites. Briefly, we calculated the analysis of variance (ANOVA) to compare the DNA methylation levels among cancer types. In ANOVA, F-statistic is the ratio of the variance among the means to the variance within the samples. F-statistic is used to measure the difference among cancers. Euclidean distance (ED) was used to measure the data redundancy. The criterion of MFMD is redefined as follow:

$$MFMD=\max\;\left(w_{s}\times Fstatistic+w_{d}\times ED\right)$$

the variable *w*_s_ (0 < *w*_s_ ≤1) and *w*_d_ (0 < *w*_d_ ≤1) are the weights of F-statistic and distance, respectively. We ranked the CpG sites according to the MFMD values. The final feature set will have lowest ED values and highest F-statistic values. Then, top-ranked CpG sites were selected as features to construct classifiers and evaluate the classification accuracy. The top-ranked CpG sites with highest accuracy were selected as the final features.

## Results

Comparison of the classifiers using mRNA, miRNA, lncRNA and DNA methylation profiles for the tumor tissue origin prediction.

Gene expression profiles (mRNA, miRNA and lncRNA) and DNA methylation profiles were obtained from TCGA cohort. After a strict review of these four different types of datasets, tumor samples spanning 20 cancer types were collected (Table 1). It was randomly divided into two equal parts (a training cohort and a testing cohort). For the gene expression profiles, the expression values (FPKM value) of all genes were used, and a total of 12,692 mRNA, 1,240 miRNA and 5,642 lncRNAs were enrolled. For the DNA methylation profiles, we adopted a feature selection step to select tissue-specific CpG methylation because of the high dimensionality. A total of 120,106 differentially methylated CpG sites were detected, which were distributed across the entire human genome. Then, the optimal number of principle components were determined using PCA (cumulative percentage of total variation >95%). As an outcome of dimensionality reduction process, machine learning models have been developed using 2,974 components.

**TABLE 1:** Cancer types and their respective sample size.

Cancer type	Abbreviation	Sample size
Bladder urothelial carcinoma	BLCA	380
Breast invasive carcinoma	BRCA	652
Cervical squamous cell carcinoma	CESC	259
Colorectal adenocarcinoma	COAD	358
Esophageal carcinoma	ESCA	170
Head and neck squamous cell carcinoma	HNSC	464
Kidney renal clear cell carcinoma	KIRC	279
Kidney renal papillary cell carcinoma	KIRP	237
Brain lower grade glioma	LGG	410
Liver hepatocellular carcinoma	LIHC	352
Lung adenocarcinoma	LUAD	415
Lung squamous cell carcinoma	LUSC	310
Pancreatic adenocarcinoma	PAAD	163
Pheochromocytoma and paraganglioma	PCPG	157
Prostate adenocarcinoma	PRAD	454
Sarcoma	SARC	229
Stomach adenocarcinoma	STAD	356
Testicular germ cell tumors	TGCT	137
Thyroid carcinoma	THCA	434
Uterine corpus endometrial carcinoma	UCEC	395
Total		6,738

**FIGURE 1:**
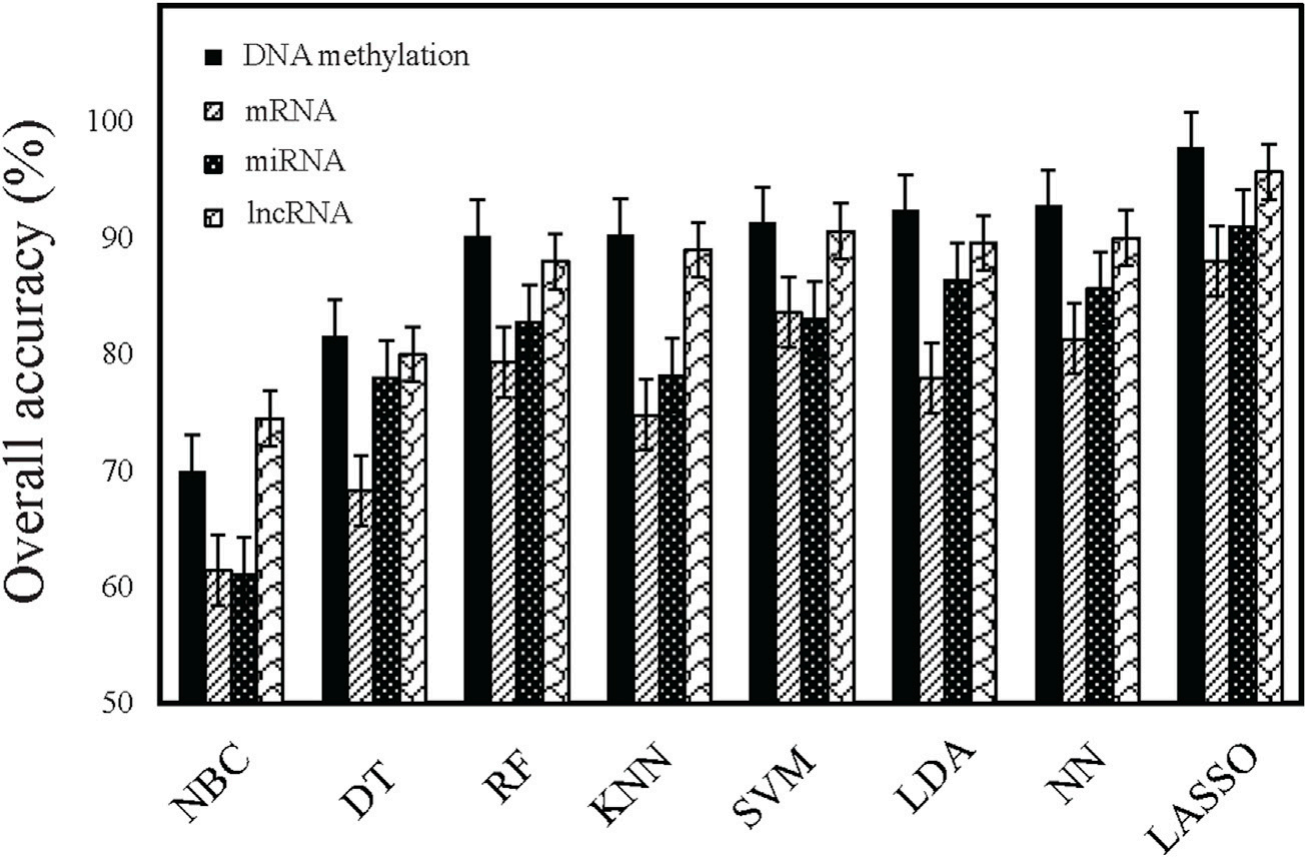
Comparison of the performance of different classifiers. Performance is estimated based on overall accuracy derived from multi-class classification tasks.

We used eight machine learning algorithms to train classifiers (see Materials and Methods). Figure 1 summarized the overall accuracy of each classifier. A comparison of the results clearly showed that most of the classifiers achieved good performance (>80%), among which LASSO is the most predictive model with the highest overall accuracy (Table 2). Consistent with previous works (Lu et al., 2005; Ma et al., 2006; Li et al., 2007; Elias et al., 2017), it has been indicated that the expression-based classifiers achieved competitive performance with the overall accuracy of 88.01% (mRNA-based), 91.03% (miRNA-based), respectively. We demonstrated that lncRNA-based profiles also achieved competitive performance for the first time (overall accuracy 95.7%). Our work indicated that DNA methylation-based classifiers (overall accuracy 97.77%) performs better than other gene expression-based classifiers.

**TABLE 2:** Precision and recall of each of the 20 cancer types using the LASSO model.

Cancer type	DNA methylation		mRNA		lncRNA		miRNA	
	Precision (%)	Recall (%)	Precision (%)	Recall (%)	Precision (%)	Recall (%)	Precision (%)	Recall (%)
Bladder urothelial carcinoma	99.47	97.63	94.33	92.70	94.73	91.24	88.59	86.16
Breast invasive carcinoma	98.83	99.59	98.64	99.09	98.46	98.72	97.72	97.89
Cervical squamous cell carcinoma	97.62	95.74	92.00	90.79	93.19	89.15	85.73	85.02
Colorectal adenocarcinoma	98.29	92.57	81.62	92.33	80.06	90.62	79.79	86.92
Esophageal carcinoma	85.19	77.06	88.87	80.15	86.55	77.63	73.79	58.69
Head and neck squamous cell carcinoma	97.70	98.92	93.09	93.60	91.12	92.20	89.20	91.58
Kidney renal clear cell carcinoma	98.26	97.48	96.57	94.19	95.09	94.00	97.46	95.77
Kidney renal papillary cell carcinoma	98.34	97.48	94.46	93.75	93.70	93.41	92.91	93.83
Brain lower grade glioma	98.08	98.78	99.23	99.22	98.26	98.63	100.00	99.81
Liver hepatocellular carcinoma	99.43	98.86	97.60	95.96	97.30	96.49	98.63	97.05
Lung adenocarcinoma	96.27	98.80	94.25	93.52	91.41	93.13	91.73	93.40
Lung squamous cell carcinoma	97.01	93.87	89.14	90.82	85.70	87.42	86.29	85.36
Pancreatic adenocarcinoma	100.00	100.00	93.11	95.44	89.63	96.03	87.92	93.78
Pheochromocytoma and paraganglioma	100.00	100.00	100.00	97.76	100.00	98.32	99.46	98.30
Prostate adenocarcinoma	100.00	100.00	100.00	99.80	99.80	99.40	97.47	100.00
Sarcoma	99.13	96.95	91.32	91.89	92.23	91.52	93.84	94.21
Stomach adenocarcinoma	86.27	83.54	81.60	84.14	90.38	91.73	84.81	87.84
Testicular germ cell tumors	99.33	100.00	98.71	100.00	99.35	99.33	99.35	98.67
Thyroid carcinoma	100.00	100.00	99.80	99.60	99.61	100.00	100.00	99.60
Uterine corpus endometrial carcinoma	95.10	98.23	95.56	98.35	94.31	96.90	93.81	94.64
Average accuracy (%)	97.77		88.01		95.70		91.03	

Since the overall accuracy cannot tell us how well each cancer type is classified, a 5-fold cross-validation was performed in the testing dataset. The results indicated that the classifiers do not classify all cancer types equally well (Table 2). The precision and recall values are generally high for all the cancer types. With the exception of esophageal carcinoma (precision: 85.19%, recall: 77.06%) and Stomach adenocarcinoma (precision: 86.27%, recall: 83.54%), all other cancer types have precision and recall values larger than 90% in the testing set. Notably, the precision and recall values reach to 100% for pancreatic adenocarcinoma, Pheochromocytoma and paraganglioma, thyroid carcinoma and prostate adenocarcinoma.

### Performance of classifiers using small number of CpG markers

**FIGURE 2:**
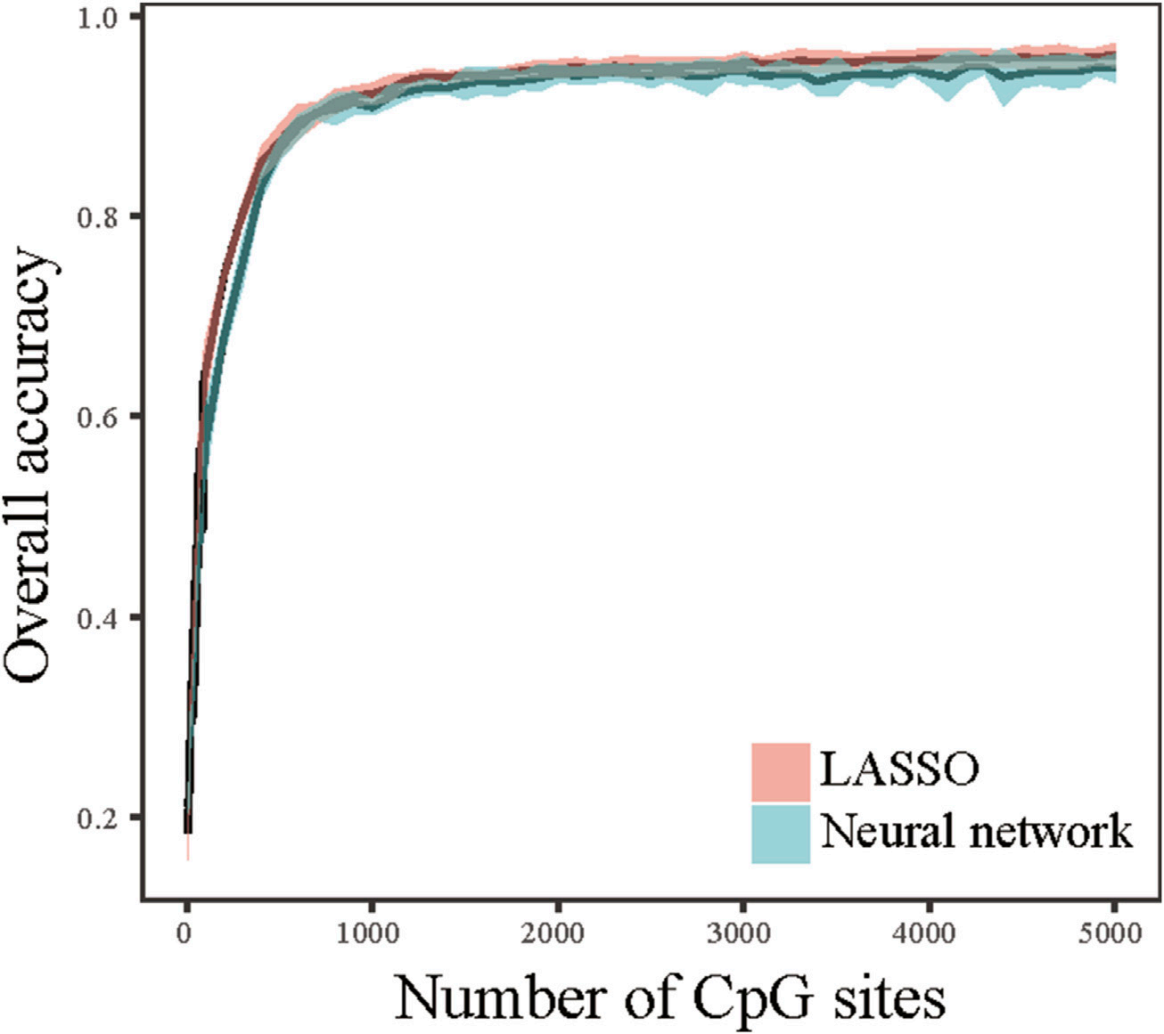
Performance of top-ranked CpG markers. The overall accuracies were calculated as a function of the number of CpG markers using LASSO and neural network classifiers.

We next attempted to determine whether tumor tissue origin can be predictive using small number CpG markers. Selecting true tumor tissue specific features is important to construct classifiers that performs well at predicting tumor origin sites. To this end, we proposed a method called Maximum-F-statistic-Maximum-Distance (MFMD) to measure the tumor tissue specificity and redundancy of CpG sites. The feature candidates were ranked based on MFMD score, and the top-ranked features were used to construct classifiers to evaluate the classification accuracy. Figure 2 summarized the performance of the classifiers as a function of the number of CpG markers. The result showed a sharp increase in the overall accuracy of the classifiers at the initial stage when the number of CpG sites is small. There are diminishing increases of overall accuracy with the involvement of additional CpG sites. When the number of CpG sites used reaches 1,000 (Supplementary Table S1), it is enough to achieve an overall accuracy >90% and the overall accuracy of the classifier starts to level off. This result indicated that a competitive performance of the classifiers can be achieved using a small number of CpG markers. We further measured the locations across the chromosome of these CpG sites, and found that most of the CpG sites are located at introns (45.7%) and promoters (21.4%).

## Discussion

Gene expression and DNA methylation profiles have become the basis for diagnosis and prognosis prediction, and are important for the detection of tumor tissue origin (Moran et al., 2016; Hao et al., 2017; Rani et al., 2017). With the emerging of high-throughput technologies, large amount of data has been generated, which provided us great importance to improve the prediction of tumor tissue origin. The goal of this work is to explore the potential and limitation of utilizing different profiles as a cancer diagnostic way.

Gene expression signature of mRNA and microRNA expression levels have been used for tumor tissue origin detection (Tothill et al., 2005; Sotiriou and Piccart, 2007; Rosenfeld et al., 2008; Elias et al., 2017). DNA methylation, microRNA and lncRNA are important class of regulatory mechanism, and are central to numerous biological processes. The comparison of eight benchmark machine learning based classifiers demonstrated that LASSO model is the best choice, and reached overall accuracy of >90% in 20 cancer types. Using large number of features may bring dome degree of over-fitting of the classifier, we divided the TCGA data into a training set and testing set. The 5-fold cross-validation further indicated that our prediction has high precision and recall values in each cancer type prediction. Comparison of the performance of classifiers based on different profiles is necessary. The classifiers for DNA methylation-, mRNA-, microRNA- and lncRNA-based are all demonstrated promising results on predicting tumor tissue origin. Here, we tried to quantify the performance using mRNA-based, miRNA-based, lncRNA-based and DNA methylation-based profiles to identify cancer type. A more competitive performance of DNA methylation-based classifiers was obtained than mRNA-, microRNA- and lncRNA-based classifiers. Here, we demonstrated that the expression profiles of lncRNAs can accurately identify tumor tissue origin for the first time. Because archival formalin-fixed paraffin-embedded (FFPE) samples are important source for tumor material, the application is limited by its instability of lncRNA in FFPE samples. DNA methylations have several features that make them attractive diagnostic biomarkers. First, DNA methylation shows better stability and can largely maintain its methylated status in archival FFPE samples. Second, DNA methylation shows marked tissue specificity, and plays a key role in embryonic development. In this regards, differentially methylated CpG sites would be enriched for tissue-specific markers, and would provide a starting point for the development of tumor tissue origin classifier.

## Conclusion

Taken together, we have showed that LASSO classifier can efficiently predict tumor tissue origin based on DNA methylation profiles. Moreover, the performance of DNA methylation-based classifiers is better than that of gene expression-based classifiers. Our results demonstrated the effectiveness of DNA methylation profiles as biomarkers for the prediction of tumor tissue origin.

## Data Availability

The datasets presented in this study can be found in online repositories. The names of the repository/repositories and accession number(s) can be found in the article/Supplementary Material.

## References

[B1] Babak T. Zhang W. Morris Q. Blencowe B. J. Hughes T. R. (2004). Probing microRNAs with microarrays: tissue specificity and functional inference. RNA 10, 1813–1819. 10.1261/rna.7119904 15496526 PMC1370668

[B2] Bloom G. Yang I. V. Boulware D. Kwong K. Y. Coppola D. Eschrich S. (2004). Multi-platform, multi-site, microarray-based human tumor classification. Am. J. Pathol. 164, 9–16. 10.1016/S0002-9440(10)63090-8 14695313 PMC1602228

[B3] Cerami E. Gao J. Dogrusoz U. Gross B. E. Sumer S. O. Aksoy B. A. (2012). The cBio cancer genomics portal: an open platform for exploring multidimensional cancer genomics data. Cancer Discov. 2, 401–404. 10.1158/2159-8290.CD-12-0095 22588877 PMC3956037

[B4] Ehrlich M. (2002). DNA methylation in cancer: too much, but also too little. Oncogene 21, 5400–5413. 10.1038/sj.onc.1205651 12154403

[B5] Elias K. M. Fendler W. Stawiski K. Fiascone S. J. Vitonis A. F. Berkowitz R. S. (2017). Diagnostic potential for a serum miRNA neural network for detection of ovarian cancer. Elife 6, e28932. 10.7554/eLife.28932 29087294 PMC5679755

[B6] Hainsworth J. D. Greco F. A. (1993). Treatment of patients with cancer of an unknown primary site. N. Engl. J. Med. 329, 257–263. 10.1056/NEJM199307223290407 8316270

[B7] Hao X. Luo H. Krawczyk M. Wei W. Wang W. Wang J. (2017). DNA methylation markers for diagnosis and prognosis of common cancers. Proc. Natl. Acad. Sci. U. S. A. 114, 7414–7419. 10.1073/pnas.1703577114 28652331 PMC5514741

[B8] Kang S. Li Q. Chen Q. Zhou Y. Park S. Lee G. (2017). CancerLocator: non-invasive cancer diagnosis and tissue-of-origin prediction using methylation profiles of cell-free DNA. Genome Biol. 18, 53. 10.1186/s13059-017-1191-5 28335812 PMC5364586

[B9] Lagos-Quintana M. Rauhut R. Yalcin A. Meyer J. Lendeckel W. Tuschl T. (2002). Identification of tissue-specific microRNAs from mouse. Curr. Biol. 12, 735–739. 10.1016/s0960-9822(02)00809-6 12007417

[B10] Li J. Smyth P. Flavin R. Cahill S. Denning K. Aherne S. (2007). Comparison of miRNA expression patterns using total RNA extracted from matched samples of formalin-fixed paraffin-embedded (FFPE) cells and snap frozen cells. BMC Biotechnol. 7, 36. 10.1186/1472-6750-7-36 17603869 PMC1914054

[B11] Lu J. Getz G. Miska E. A. Alvarez-Saavedra E. Lamb J. Peck D. (2005). MicroRNA expression profiles classify human cancers. Nature 435, 834–838. 10.1038/nature03702 15944708

[B12] Ma X. J. Patel R. Wang X. Salunga R. Murage J. Desai R. (2006). Molecular classification of human cancers using a 92-gene real-time quantitative polymerase chain reaction assay. Arch. Pathol. Lab. Med. 130, 465–473. 10.1043/1543-2165(2006)130[465:MCOHCU]2.0.CO;2 16594740

[B13] Moran S. Martinez-Cardus A. Sayols S. Musulen E. Balana C. Estival-Gonzalez A. (2016). Epigenetic profiling to classify cancer of unknown primary: a multicentre, retrospective analysis. Lancet Oncol. 17, 1386–1395. 10.1016/S1470-2045(16)30297-2 27575023

[B14] Oien K. A. (2009). Pathologic evaluation of unknown primary cancer. Semin. Oncol. 36, 8–37. 10.1053/j.seminoncol.2008.10.009 19179185

[B15] Paz M. F. Fraga M. F. Avila S. Guo M. Pollan M. Herman J. G. (2003). A systematic profile of DNA methylation in human cancer cell lines. Cancer Res. 63, 1114–1121. 12615730

[B16] Pimiento J. M. Teso D. Malkan A. Dudrick S. J. Palesty J. A. (2007). Cancer of unknown primary origin: a decade of experience in a community-based hospital. Am. J. Surg. 194, 833–837. 10.1016/j.amjsurg.2007.08.039 18005780

[B17] Ramaswamy S. Tamayo P. Rifkin R. Mukherjee S. Yeang C. H. Angelo M. (2001). Multiclass cancer diagnosis using tumor gene expression signatures. Proc. Natl. Acad. Sci. U. S. A. 98, 15149–15154. 10.1073/pnas.211566398 11742071 PMC64998

[B18] Rani L. Mathur N. Gupta R. Gogia A. Kaur G. Dhanjal J. K. (2017). Genome-wide DNA methylation profiling integrated with gene expression profiling identifies PAX9 as a novel prognostic marker in chronic lymphocytic leukemia. Clin. Epigenetics 9, 57. 10.1186/s13148-017-0356-0 28572861 PMC5450117

[B19] Rosenfeld N. Aharonov R. Meiri E. Rosenwald S. Spector Y. Zepeniuk M. (2008). MicroRNAs accurately identify cancer tissue origin. Nat. Biotechnol. 26, 462–469. 10.1038/nbt1392 18362881

[B20] Schubeler D. (2015). Function and information content of DNA methylation. Nature 517, 321–326. 10.1038/nature14192 25592537

[B21] Shen S. Wang G. Shi Q. Zhang R. Zhao Y. Wei Y. (2017). Seven-CpG-based prognostic signature coupled with gene expression predicts survival of oral squamous cell carcinoma. Clin. Epigenetics 9, 88. 10.1186/s13148-017-0392-9 28852427 PMC5571486

[B22] Soh K. P. Szczurek E. Sakoparnig T. Beerenwinkel N. (2017). Predicting cancer type from tumour DNA signatures. Genome Med. 9, 104. 10.1186/s13073-017-0493-2 29183400 PMC5706302

[B23] Sotiriou C. Piccart M. J. (2007). Taking gene-expression profiling to the clinic: when will molecular signatures become relevant to patient care? Nat. Rev. Cancer 7, 545–553. 10.1038/nrc2173 17585334

[B24] Staub E. Buhr H. J. Grone J. (2010). WITHDRAWN: predicting the site of origin of tumors by a gene expression signature derived from normal tissues. Oncogene 29, 4485–4492. 10.1038/onc.2009.398 20514016

[B25] Stieglitz E. Mazor T. Olshen A. B. Geng H. Gelston L. C. Akutagawa J. (2017). Genome-wide DNA methylation is predictive of outcome in juvenile myelomonocytic leukemia. Nat. Commun. 8, 2127. 10.1038/s41467-017-02178-9 29259179 PMC5736624

[B26] Tang W. Wan S. Yang Z. Teschendorff A. E. Zou Q. (2017). Tumor origin detection with tissue-specific miRNA and DNA methylation markers. Bioinformatics 34, 398–406. 10.1093/bioinformatics/btx622 29028927

[B27] Tothill R. W. Kowalczyk A. Rischin D. Bousioutas A. Haviv I. Van Laar R. K. (2005). An expression-based site of origin diagnostic method designed for clinical application to cancer of unknown origin. Cancer Res. 65, 4031–4040. 10.1158/0008-5472.CAN-04-3617 15899792

[B28] Varadhachary G. R. Spector Y. Abbruzzese J. L. Rosenwald S. Wang H. Aharonov R. (2011). Prospective gene signature study using microRNA to identify the tissue of origin in patients with carcinoma of unknown primary. Clin. Cancer Res. 17, 4063–4070. 10.1158/1078-0432.CCR-10-2599 21531815

[B29] Wang Y. Wu N. Liu J. Wu Z. Dong D. (2015). FusionCancer: a database of cancer fusion genes derived from RNA-seq data. Diagn Pathol. 10, 131. 10.1186/s13000-015-0310-4 26215638 PMC4517624

[B30] Xu Q. Chen J. Ni S. Tan C. Xu M. Dong L. (2016). Pan-cancer transcriptome analysis reveals a gene expression signature for the identification of tumor tissue origin. Mod. Pathol. 29, 546–556. 10.1038/modpathol.2016.60 26990976

[B31] Yang Z. Wu L. Wang A. Tang W. Zhao Y. Zhao H. (2017). dbDEMC 2.0: updated database of differentially expressed miRNAs in human cancers. Nucleic Acids Res. 45, D812–D818. 10.1093/nar/gkw1079 27899556 PMC5210560

[B32] Zheng G. Ma Y. Zou Y. Yin A. Li W. Dong D. (2018). HCMDB: the human cancer metastasis database. Nucleic Acids Res. 46, D950–D955. 10.1093/nar/gkx1008 29088455 PMC5753185

